# Three-Dimensional Cell Cultures as an In Vitro Tool for Prostate Cancer Modeling and Drug Discovery

**DOI:** 10.3390/ijms21186806

**Published:** 2020-09-16

**Authors:** Fabrizio Fontana, Michela Raimondi, Monica Marzagalli, Michele Sommariva, Nicoletta Gagliano, Patrizia Limonta

**Affiliations:** 1 Department of Pharmacological and Biomolecular Sciences, Università degli Studi di Milano, via Balzaretti 9, 20133 Milan, Italy; michela.raimondi@unimi.it (M.R.); monica.marzagalli@unimi.it (M.M.); patrizia.limonta@unimi.it (P.L.); 2 Department of Biomedical Sciences for Health, Università degli Studi di Milano, via Mangiagalli 31, 20133 Milan, Italy; michele.sommariva@unimi.it (M.S.); nicoletta.gagliano@unimi.it (N.G.)

**Keywords:** prostate cancer, cell culture, 2D, bilayer, 3D, spheroid, animal model, cell signaling, drug discovery, drug screening

## Abstract

In the last decade, three-dimensional (3D) cell culture technology has gained a lot of interest due to its ability to better recapitulate the in vivo organization and microenvironment of in vitro cultured cancer cells. In particular, 3D tumor models have demonstrated several different characteristics compared with traditional two-dimensional (2D) cultures and have provided an interesting link between the latter and animal experiments. Indeed, 3D cell cultures represent a useful platform for the identification of the biological features of cancer cells as well as for the screening of novel antitumor agents. The present review is aimed at summarizing the most common 3D cell culture methods and applications, with a focus on prostate cancer modeling and drug discovery.

## 1. Introduction

Prostate cancer (PCa) represents the second leading cause of tumor mortality among men in Western countries [1]. In recent years, considerable efforts have been made to identify the molecular mechanisms underlying its development and progression as well as to define novel approaches for its treatment [2,3]. In this setting, besides the canonical in vitro and in vivo studies, several experiments have been conducted by exploiting new three-dimensional (3D) cell culture technologies, which not only provide a deeper understanding of PCa biology, but also offer fundamental insights about PCa drug response in a cost/time effective and high throughput way [4]. Considering these advantages, this review article is aimed at describing the existing 3D PCa cell culture systems and at discussing their crucial role in tumor modeling and drug discovery.

## 2. 3D Cell Cultures as Preclinical Models of PCa

Cell culture represents one of the basic techniques used to study PCa biology. Traditional cell culture methods involve the use of PCa cell lines, including the well-known LNCaP, PC3, and DU145 cells, grown in a two-dimensional (2D) monolayer [5]. However, due to their inherent flaws, 2D cultures fail to properly mimic the in vivo tumor architecture and microenvironment, showing fundamental differences in cell morphology, proliferation, differentiation, metabolism, and signal transduction [6,7]. On the other hand, despite being essential to monitor drug bioavailability, therapeutic efficacy, and dose-limiting toxicity, animal models, including the well-established TRAMP and LADY mice, present a number of problems, such as higher costs, species differences, limited availability, and feasibility [5,8]. Furthermore, ethical issues about the use of animals in cancer research are highly controversial, and animal testing is strongly discouraged if it can be replaced with cell culture-based assays [9,10]. To overcome these limitations, different 3D PCa cell culture methods have been developed. The common objective of these models is to recreate the in vivo organization of the tumor, serving as powerful tools for the study of both PCa biology and drug response and thus bridging the gap between the conventional 2D cultures and mice [11]. An overview of the main techniques and applications of 3D PCa cell culture is presented in the following paragraphs.

### Methods of 3D PCa Cell Culture

3D PCa cell cultures can be obtained by using different technologies.

A very popular method to grow PCa spheroids involves their self-assemblance in non-adherent culture conditions, where tumor cells are forced to aggregate on attachment-limiting surfaces generally coated with agarose. Despite being relatively simple, low-cost, and reproducible and offering great potential for the study of cell–cell interactions, this approach presents important problems with drug testing as no accurate drug response or cell viability assay has been developed so far [12,13,14]. Interestingly, in a series of studies by O’Connor et al., Smoluchowski’s population balance equation and Monte Carlo simulation have been used to explore the self-adhesion dynamics and predict the aggregation kinetics of DU145 and LNCaP cells in liquid-overlay cultures, proving to be particularly suitable for the identification of the proper culture conditions for the production of highly viable spheroids for in vitro tissue regeneration [15,16,17,18,19].

PCa tumoroids can also be easily obtained by the hanging drop technique, in which cancer cells are seeded and incubated in hanging drops until they form rounded structures characterized by stable cell–cell contacts. The starting cell numbers as well as the required media volumes are small, and spheroid size can be controlled by increasing/reducing the days of culture. More importantly, this method provides the opportunity to co-culture tumor cells with other cell populations to study the effect of their cross-talk. However, after initiation of the cell culture, it is not possible to replace the media, and chemicals and pharmacological agents can hardly be added in the middle of the drop, significantly limiting the use of this system in drug discovery [20,21].

A more complex approach is represented by the organ chip technology, based on microfluidic devices made of plastic, glass or flexible polymers containing hollow microchannels where PCa cells are grown to recreate the in vitro architecture of the in vivo tumor mass [22]. Notably, other types of cells can also be included in these devices, allowing the dissection of PCa microenvironment, especially in relation to tumor progression and metastasis. In this regard, Hsiao et al. have recently described the design and fabrication of a platform for efficient microfluidic 3D co-culture of PC3 cells within a niche-like construct composed of osteoblasts and endothelial cells. Besides promoting the generation of uniform-sized spheroids, this system ensures equal distribution of the three cell types across all aggregates, keeping them still during media exchange and thus facilitating long-term cultures [23].

As it is well-known that extracellular matrix affects cellular organization and tissue functions, novel 3D PCa models incorporating extracellular matrix-like gels have been developed. These substrates are generally highly hydrophilic polymers with a soft tissue-like stiffness designed to mimic the extracellular protein network and include Matrigel, alginate, and collagen. PCa cells cultured in the gel usually aggregate spontaneously, giving rise to a tumoroid that exhibits not only cell–cell adhesions, but also cell–artificial extracellular matrix contacts [24,25,26,27,28,29].

An additional anchorage-independent 3D PCa environment can be established by utilizing prefabricated scaffolds, which function as surrogates for the missing extracellular matrix and provide the physical support for spheroid formation and growth. These scaffolds consist of porous materials derived from natural (i.e., collagen and chitosan) or synthetic (i.e., polycaprolactone) polymers: the first have lower toxicity and improved biocompatibility, while the second demonstrate enhanced workability, reproducibility, and versatility. In general, the procedures for scaffold preparation are much more complicated and expensive than those for gel production; however, they ensure maximum resemblance to the in vivo conditions [30,31,32].

The use of immortalized PCa cell lines to test the efficacy of new drugs in vitro or in vivo is economically advantageous, but also responsible for the failure of several molecules entering clinical trials. Based on these premises, an alternative, promising approach consisting of the ex vivo 3D culture of freshly excised PCa specimens, called patient-derived explants (PDEs), has been recently proposed. This protocol, which involves the growth of tissue pieces or slices on sponge scaffolds, offers a cost-effective model that recalls the native tumor mass architecture and allows the direct assessment of treatment responses on an individual patient’s sample, enabling the development of personalized medicine strategies [33,34,35,36,37,38].

The existing methods of 3D PCa cell culture, together with their main advantages and disadvantages, are schematically represented in Table 1.

## 3. Cellular Signaling in 3D PCa Models

**Figure 1:**
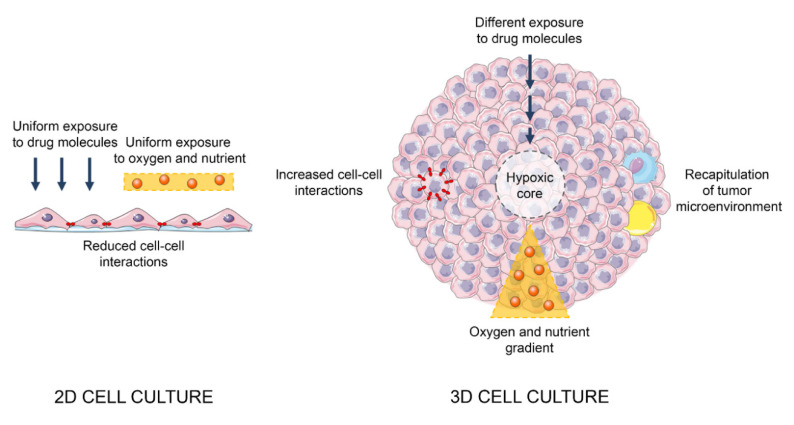
Main differences between 2D and 3D cell cultures.

In the last twenty years, 3D models have shown great potential for the functional study of different malignancies. In particular, the improvement and consolidation of novel 3D culture techniques, as well as the wide characterization of an extensive range of cancer cell lines, has offered to researchers the possibility to explore the cellular pathways implicated in tumor development and progression. Indeed, cancer spheroids have been demonstrated to exhibit tumor tissue-like biological features and, when coupled with stromal and immune cells, to recapitulate the in vivo microenvironment, proving to be more biologically relevant than their 2D counterparts [4,11] (Figure 1). As described in the following section, multiple cellular and molecular mechanisms underlying PCa behavior have been assessed and elucidated in 3D settings.

### 3.1. Extracellular Matrix Remodeling and Epithelial-to-Mesenchymal Transition

A significant amount of research into PCa cellular and molecular alterations has been conducted using 3D cultures, including analysis of the variations at both genetic and protein levels between different cell culture conditions. In an interesting study by Härmä et al., gene expression of a panel of 29 prostate tumor and non-tumor cell lines has been compared between 2D and 3D models [24]. Significant expression changes were detected for genes involved in extracellular matrix (ECM) remodeling and epithelial-to-mesenchymal transition (EMT), with PCa tumoroids showing an upregulation of integrins, such as ITGB2, ITGB4, and ITGA10, and epithelial markers, including laminin-5 and connexin 43. In this regard, it should be noted that primary and non-transformed prostate epithelial cells formed well-differentiated round spheroids, with stable cell–cell contacts, epithelial polarization and a hollow lumen, while most PCa cell lines formed large, poorly differentiated and highly invading aggregates [24]. Similar results were also obtained by Fontana et al., who highlighted the inability of metastatic, castration-resistant PC3 cells to give rise to well-organized 3D structures [14]. Intriguingly, also in this case, the re-expression of epithelial markers (i.e., E-cadherin) and the parallel downregulation of mesenchymal proteins (i.e., vimentin, Slug, Twist, and Zeb1) were found in 3D PCa cell cultures, suggesting that the pattern of invasion observed during acinar formation could be representative of a streaming or chain migration rather than of the acquisition of a clear and univocal mesenchymal phenotype [14]. This hypothesis has also been confirmed by recent evidence showing that spheroid forming capacity of BPH-1 cells is significantly abrogated after stable transduction of the EMT-promoting transcription factor Snail as well as after transfection of metastasis-inducing membrane type 1 matrix metalloproteinase (MT1-MMP) [39,40]. Notably, spheroidogenic ability of PCa cell lines has been reported to be crucially linked to lipid metabolism, specifically to lysophosphatidic acid and sphingosine-1-phosphate signaling [41]. Collectively, these results highlight the dynamic nature and plasticity of PCa 3D models as well as their fundamental role in the study of tumor metastatic behavior, balancing cancer cell growth and invasion and thus reproducing the mechanisms of both tumor initiation and spread.

### 3.2. Evaluation of Clinical and Diagnostic Features of PCa

Prostate cells produce prostate-specific antigen (PSA), and serum PSA level is universally recognized as a biomarker of PCa [42]. PSA concentration has been measured in the medium of 3D LNCaP cell cultures: after one week it reached its peak, corresponding to spheroid growth progression with time. In addition, PSA levels were decreased by oxaliplatin treatment, demonstrating a correlation with spheroid survival [43]. On the other hand, analysis of PSA expression in 3D PC3 models has given controversial results. While PSA protein was detected in PC3 cells grown in Matrigel but not in monolayers on plastic, RT-PCR data indicated that 3D growth downregulated PSA expression at the transcriptional level. This effect may be the consequence of a PSA gene feedback repression by PSA protein accumulated in Matrigel-cultured cells, while PSA produced by PC3 cells in monolayers may be quickly secreted into the supernatant [44]. Interestingly, mixing culture medium significantly downregulated PSA expression and spatial organization in DU145 spheroids, leading to tumor cell dedifferentiation presumably caused by improved interstitial transport and synthesis of extracellular matrix [45]. Many experiments are ongoing to further investigate if common PCa cell lines cultivated with 3D methods can retain the clinical/diagnostic features of the original tumor.

### 3.3. Androgen Signaling

Human prostate releases large amounts of nerve growth factor (NGF) [46], which, in turn, regulates the physiological development of the gland by binding to the tropomyosin receptor kinase A (TrkA) [47]. This signaling is severely deregulated in PCa. Indeed, in androgen-dependent LNCaP cells, TrkA has been found to modulate NGF proliferative effects via interaction with the androgen receptor (AR) [48]. Remarkably, once activated by NGF, TrkA can also stimulate the growth and invasiveness of a variety of androgen-independent PCa cell lines [49]. In agreement with these observations, the NGF/TrkA axis has been recently reported to promote the acquisition of a more aggressive phenotype in different miniaturized 3D PCa systems, suggesting that targeting of this cascade might be of value in the clinical approach of both hormone-driven and -refractory malignancies [49].

### 3.4. Assessing Tumor Hypoxia

Within cancer spheroids, oxygen consumption rates differ substantially from those registered during 2D growth, where a larger surface area-to-volume ratio exists, and all the cells have equal contact with the supernatant. Indeed, in 3D models only the outer layer of cells directly interacts with the medium, simulating the in vivo hypoxic conditions where only a small portion of tumor tissue is reached by blood vessels [50]. Tagaki et al. have shown an enhanced production of vascular endothelial growth factor (VEGF) in 3D LNCaP cell cultures, suggesting that it might depend on the hypoxic state within the tumoroids. In particular, hypoxia-inducible transcriptional mediator 1 (HIF-1)-induced VEGF may be used to enable the survival of the spheroid via an autocrine mechanism and to delay the onset of necrotic cell death in the core of the aggregate [51]. Whether such pathways are evoked in other 3D PCa models remains to be verified.

### 3.5. Modeling Cancer Stem Cell Plasticity

Current cancer stem cell (CSC) theory states that tumor cells are hierarchically organized, with CSCs representing a small fraction of the bulk cells and being capable of generating the entire tumor mass due to their potential for self-renewal and differentiation [52]. However, owing to the plasticity of CSC-related phenotypes, the identification of these progenitor cells from solid tumors remains difficult. As a result, CSC characterization relies on cell-surface markers and on in vivo tumor-formation assays after transplantation into immunodeficient mice. General procedures for the in vitro expansion of CSCs are based on their unique ability to survive and proliferate as spheres in serum-free medium [53]. 3D multicellular tumor cultures are typically established from cancer cell lines in conventional medium supplemented with FBS, like classical monolayers. However, they can also serve as surrogate systems to evaluate CSC-related characteristics in vitro. For instance, CD133+/CD44+ tumor-initiating cells have been isolated from DU145 cell line, propagated as spheroids and compared with those obtained by aggregation of the heterogeneous bulk population. Surprisingly, downregulation of WNT1-related pathways (i.e., FZD1, APC, ADAR, and PPARD) and upregulation of TGF-β-associated signaling (i.e., VCAN, COL7A1, and ITGβ3) have been found in CSC tumoroids when compared to non-CSC acini, supporting a 3D model where cancer progenitor cells are engaged by different molecular cascades to differentiate and initiate tumor formation [54,55]. Conversely, 3D porous chitosan–alginate scaffolds have been demonstrated to promote CSC enrichment in TRAMP-C2 cell line, with spheroids showing reduced proliferation and enhanced expression of CD133 and NANOG, as opposed to monolayers [56]. Based on this evidence, a deeper molecular characterization of cells cultured in 3D conditions is expected to clarify the in vivo significance of PCa cell stemness in the very next future.

### 3.6. Cell Redox Homeostasis and Energetics

Development of P-glycoprotein-mediated multidrug resistance (MDR) is a major cause of reduced therapy efficacy in tumors. P-glycoprotein activation after chronic treatment of cancer cells with chemotherapeutics has been extensively studied [57]. In a series of reports by Wartenberg et al., P-glycoprotein expression in 3D multicellular PCa cultures has been associated with the appearance of quiescent cell areas in large tumor spheroids, whereas small, highly proliferative aggregates showed only weak expression of this protein and consequently do not exhibit an MDR phenotype [58,59,60,61]. In particular, activation of P-glycoprotein occurred in tumoroids expressing high levels of cyclin-dependent kinase inhibitors p27Kip1 and p21WAF-1 and low levels of mitogen-activated kinases (MAPKs), including c-Jun amino-terminal kinase (JNK), extracellular signal-regulated kinase (ERK1 and 2), and p38 [58,59,60,61]. Interestingly, pro-oxidant agents, such as hydrogen peroxide, glyceraldehyde, menadione, and buthionine sulfoximine, successfully downregulated P-glycoprotein expression and reversed the MDR characteristics, but these effects were counteracted by agents interfering with receptor tyrosine kinase signaling pathways, such as bisindolylmaleimide I, Ro-31-8220, ZM 336372, and PD98059, suggesting that reactive oxygen species (ROS) involved as second messengers in the Ras/Raf/MEK/ERK cascade may act as negative regulators of P-glycoprotein activity [58,59,60,61]. Moreover, exogenous addition of pyruvate to spheroid medium significantly reduced ROS generation and increased P-glycoprotein expression, which was conversely suppressed by iodoacetate or 2-deoxy-d-glucose; these findings indicate that MDR phenotype of PCa tumoroids is closely related to the glycolytic metabolism of cancer cells and can be abolished by glycolysis inhibitors via mechanisms that involve changes in the cellular redox state [62]. The crucial role of ROS in the modulation of P-glycoprotein expression in 3D PCa cell cultures is also supported by the downregulation of this protein in Nox-1-overexpressing spheroids [63]. In summary, we can conclude that the use of 3D models has demonstrated promising potential in dissecting the molecular basis of MDR in PCa, providing new insight into its modulation and targeting.

Cell redox homeostasis is determined not only by ROS production, but also by nitric oxide (NO) generation. The interaction between ROS and NO is well described: the latter interacts rapidly and irreversibly with superoxide (O_2_^−^) to yield peroxynitrite (ONOO^−^), thereby restricting the half-life, diffusion distance, and bioactivity of free radicals in tissues [64]. It has been recently demonstrated that endothelial nitric oxide synthase (eNOS) expression and subsequent NO production are elevated in small DU145 multicellular spheroids, correlating with the generation of high levels of ROS; on the other hand, they are downregulated in parallel with the reduction of oxidative stress observed in large, quiescent aggregates. In particular, inhibition of NO generation in small tumoroids via either eNOS inhibition (by N-omega-amino-l-arginine) or specific radical scavenging (by 2-(4-carboxyphenyl)-4,4,5,5-tetramethyl imidazoline-1-oxyl 3-oxide) resulted in caspase 3 cleavage and induction of apoptosis, suggesting that NO counterbalances intracellular ROS to help tumor cells to escape from apoptotic cell death [65]. If verified in other 3D PCa models, these data might support the notion that NO/ROS balance plays a pivotal role in determining cancer cell fate.

Adenosine 5′-triphosphate (ATP) has been demonstrated to be actively secreted by cells, therefore evoking Ca^2+^-dependent signal transduction cascades in an autocrine and paracrine way [66]. By using a direct current (DC) electrical field, Sauer et al. have shown that ATP released by 3D PCa cell cultures can activate purinergic receptors and elicit a Ca^2+^ wave propagating through the spheroid, stimulating tumor growth via ROS-mediated activation of c-Fos [67,68,69,70]. These findings were also confirmed by the purinergic receptor-mediated activation of ERK1/2 downstream target p90RSK and by the subsequent increased proliferation observed after spheroid incubation with exogenous ATP [71,72]. Taken together, these reports offer a starting point for more mechanistic studies that may further clarify how ATP modulates PCa tissue growth and how to ultimately interfere with this process.

### 3.7. Inflammation

Cyclooxygenase (COX)-2-produced prostaglandin E2 (PGE2) promotes the growth of a spectrum of solid tumors [73]. However, the use of COX-2-inhibiting non-steroidal anti-inflammatory drugs (NSAIDs) in cancer therapy is limited by their well-known gastrointestinal and cardiovascular toxicity [74]. In this context, targeting microsomal PGE synthase 1 (mPGES-1), the downstream enzyme in the COX-2-dependent pathway of PGE2 synthesis, has been proposed as a new, attractive therapeutic strategy for PCa. Indeed, mPGES-1 has been found to be upregulated in 3D DU145 cell cultures with respect to monolayers, and both its knockdown and pharmacological inhibition have been shown to successfully block spheroid formation. Mechanistically, it has been observed that necrotic cells in the tumoroid can induce COX-2 and mPEGS-1 mRNA expression in live cells, thus enhancing their PGE2 secretion and sustaining PGE2-dependent PCa growth [75].

### 3.8. Angiogenesis

As previously mentioned, tumor cells cultured in physiologically relevant 3D matrices are able to recapture several essential characteristics of native malignant tissues, including angiogenesis. LNCaP cells grown in a bilayer system containing heparin-decorated, hyaluronic acid-based hydrogel particles releasing heparin-binding epidermal growth factor-like growth factor (HB-EGF) give rise to spherical tumoroids, establish strong E-cadherin-mediated cell–cell contacts, and exhibit cortical organization of F-actin, while those plated as monolayers adopt a spread-out phenotype. More importantly, the spheroids significantly increase the expression of two proangiogenic factors, VEGF-165 and interleukin-8 (IL-8), at both mRNA and protein levels [76]. Similarly, PC3 cells seeded in matrix metalloproteinase-degradable biohybrid poly (ethylene) glycol-heparin hydrogels presenting the peptide motifs GFOGER (collagen I) or IKVAV (laminin-111) show an enhanced proliferation and endothelial cell infiltration compared to non-functionalized controls [77]. Thus, these novel engineered 3D models have the potential to be used for the dissection of biological processes relevant to early PCa progression, such as tumor neovascularization.

### 3.9. Metastasis

PCa primarily metastasizes to bones [78]. When studying this mechanism, one of the main limitations is represented by the complex nature of the original bone environment and by the lack of simple, cheap, and reliable models that closely reflect the biological events occurring in human subjects [79]. In this context, Salamanna et al. have developed an in vitro 3D bone metastasis model by co-culturing human PCa cells and patient-derived bone tissue within a rolling apparatus system in either normoxic or hypoxic conditions [80]. Gene expression, Western blot, immunohistochemical, histological, and four-dimensional (4D) micro-CT analyses showed an impressive specificity of PCa cells for bone colonization and ingrowth, thereby highlighting the intrinsic osteotropism of this tumor and the need to deepen the current knowledge on this phenomenon [80]. In addition, LNCaP cells have been shown to upmodulate several genes implicated in steroid biogenesis when grown in a 3D tissue-engineered bone construct in which they can directly communicate with osteoblasts, elucidating an important molecular step in the multistage process of PCa bone colonization [81]. Notably, two more 3D culture approaches have been recently established to study the interaction between PCa cells and bone marrow adipocytes, showing that under 3D conditions metabolic interactions between cancer cells and adipose tissue differ substantially from those observed in 2D cultures [82]. Globally, the results obtained from these experiments support the application of these models in preclinical studies on PCa bone metastases, presenting the unique advantage of following the replacing/reducing/refining 3R principles for animal use.

### 3.10. PCa and Immunosurveillance

Accumulating evidence suggests that inflammation may have a key role in prostatic carcinogenesis [83]. In this respect, Dang et al. have recently used a co-culture of RAW 264.7 macrophages and PZ-HPV-7 normal prostate epithelial cells to evaluate whether macrophage-mediated inflammation could affect non-tumor prostate cell behavior. PZ-HPV-7 cells were plated on Matrigel in μ-slides, while macrophages were seeded in a separated chamber of the culture system in order to allow the communication between the two cell types only through soluble factors: an increase in prostate cell proliferation was observed and ascribed to the release of CCL3, IL-1ra, osteopontin, macrophage colony-stimulating factor 1 (M-CSF1), and glial cell-derived neurotrophic factor (GDNF) by immune cells. In particular, the uptake of these immune-related factors by PZ-HPC-7 cells was accompanied by the activation of both AKT and ERK pathways [84]. Therefore, by using a 3D culture method, the authors have provided a mechanistic explanation of how inflammation could drive prostatic disorders.

Tumor-derived extracellular vesicles (EVs) have emerged as crucial modulators of cancer-host communication, and they have been demonstrated to exert numerous functional effects on the immune system [85,86]. In most of the experiments performed so far, EVs have been purified from tumor cell culture media or patients’ plasma and added to the immune cell cultures. In such a setting, the physiological relevance of the chosen EV concentration as well as the impact of both the EV isolation method and the administration timing on results are unknown. Recently, a 3D heterotypic spheroid model composed of PC3 cells producing GFP-tagged EVs (PC3-CD63-GFP) and human peripheral blood mononuclear cells (PBMCs) has been developed, providing the opportunity to study the EV-mediated interactions between PCa and human immune cells at conditions mimicking the tumor microenvironment. In particular, flow cytometry and fluorescence imaging have shown that GFP-tagged EVs can interact with a large fraction of B cell population and remain bound at the cell surface, while only 15.7–24.1% of CD3^+^ T cells and 0.3–5.8% of CD8^+^ T cells were GFP positive [87].

In conclusion, the above studies highlight the differences occurring in both gene and protein expression profiles when culturing PCa cells in the more physiologically relevant 3D models compared to traditional bilayers. Furthermore, they provide interesting examples of how 3D co-cultures can be employed to explore the complex network of PCa microenvironment without resorting to animal experiments. More importantly, these findings also emphasize the importance of using PCa tumoroids to complete mechanistic studies on current therapeutics and novel drug candidates.

## 4. 3D Cell Cultures in Drug Discovery and Screening

Knowing the differences between cells cultured in 2D and 3D is fundamental when selecting the proper model for the screening of new molecular entities. Below, we report the results obtained from different 3D assays and approaches that have been used in PCa early-stage drug discovery practice (Table 2).

### 4.1. Radiotherapy

Radiation therapy is recommended for localized or locally advanced PCa [88]. Camus et al. have proposed the use of Surface Enhanced Raman Spectroscopy (SERS) to monitor the viability of 3D PC3 multicellular spheroids treated with radiotherapy and to define the best fractionation regimes for maximizing cell death, showing that this novel method for measuring intracellular redox potential and pH in 3D live cultures can actually represent a potential new platform for in vitro preclinical characterization of tumor models [89]. Absorbed dose profiles within PCa tumoroids simulating avascular micrometastases have also been analyzed for various liposome- and antibody-radionuclide combinations. Indeed, it has been shown that liposome systems able to deliver radiation to the central region of micrometastatic tumors can be easily engineered, when conjugates with the appropriate radionuclides are constructed [90]. However, the risk of damage to neighboring normal tissues still limits the radiation dose that can be used. In this regard, the potential of the cerulenin synthetic analog C75, a fatty acid synthase inhibitor, and of 17-N-allylamino-17-demethoxy geldanamycin (17AAG), a geldanamycin derivative, to sensitize PCa cells grown in 3D culture to ionizing radiation has been investigated, demonstrating a significant improvement of targeted radiotherapy [91,92].

### 4.2. Hormone Therapy

AR targeting remains the gold standard strategy for treatment of advanced PCa [93]. However, castration resistance still represents a major clinical problem [94]. To investigate the therapeutic effects of clinically used anti-androgens, a co-culture 3D model of PCa cells and cancer-associated fibroblasts (CAFs) has been developed. Notably, within the spheroids the PCa cell/CAF ratio significantly increased in time in favor of the tumor cells, thus mimicking the cancer microenvironment in vivo. Despite this loss of CAFs, the stromal cells significantly reduced the sensitivity of PCa cells to androgens as well as to bicalutamide and enzalutamide, without altering AR levels. In particular, an increased AKT expression was found in tumor cells and its inactivation by the PI3K inhibitor LY294002 successfully overcame the anti-androgen resistance of the spheroids [95]. Based on these findings, it is suggested that CAFs can influence drug response of PCa cells to current therapies and that the 3D model described herein can represent a valuable in vitro drug-testing tool.

### 4.3. Chemotherapy

Taxanes are Food and Drug Administration (FDA)-approved drugs for treating castration-resistant PCa [96]. In a recent study by Karandish et al., reduction-sensitive polymersomes presenting folic acid on the surface and containing docetaxel have been assembled. The presence of folic acid allows efficient targeting of the prostate-specific membrane antigen (PSMA) receptor and subsequent internalization of the polymeric vesicles in LNCaP spheroids, leading to a significant decrease in tumor cell viability [97]. Similarly, the micellar delivery systems obtained by conjugation of paclitaxel to a poly(ethylene glycol methyl ether acrylate)-b-poly(carboxyethyl acrylate) (POEGMEA-b-PCEA-PTX) block copolymer have shown faster and higher cytotoxicity than the free drug in 3D PCa models [98]. Given their ability to easily reach the inner core of tumor mass-resembling spheroids, both drug-encapsulating polymersomes and micelles might thus represent useful nanodrug carriers for PCa chemotherapy.

The anthracycline doxorubicin, alone or in combination with other agents, has been extensively used to treat hormone refractory PCa, but controversial results have been reported [99]. Treatment of 3D LNCaP-N3 multicellular cultures with this drug markedly reduced PSA, E-cadherin, and keratin expression, culminating in cancer cell death [100]. However, in large tumoroids doxorubicin was found to accumulate only in the peripheral proliferating cell rim, while deeper, quiescent cell layers remained unstained, due to the overexpression of P-glycoprotein in these areas [101]. To enhance the penetration of the drug in PCa spheroids and thus in tumor tissues, a conjugate containing a matrix metalloproteinase 2 (MMP-2)-responsive N-(2-hydroxypropyl)methacrylamide (HPMA) copolymer linked to a tumor-homing cyclic peptide iRGD has been recently developed [102].

### 4.4. Targeted Therapies

The phosphatidylinositol 3-kinase (PI3K)/AKT/mammalian target of rapamycin (mTOR) signaling pathway is frequently mutated in PCa, and specific inhibitors of this cascade are now in advanced clinical trials [103]. PC3 tumoroids treated with different inhibitors against PI3K, AKT, and mTOR exhibited a significant reduction in cell growth, with just a moderate alteration of lipid content but with a massive downregulation of aerobic glycolysis [104,105]. In particular, a decreased ratio of NAD^+^/NADH was observed, encouraging the use of hyperpolarized magnetic resonance spectroscopy (HP-MRS) to monitor therapeutic response. Intriguingly, treated spheroids showed reduced lactate production with on-target inhibition confirmed by immunohistochemistry, suggesting that HP-MRS can be used to test the efficacy of PI3K/AKT/mTOR inhibitors in 3D PCa cell cultures [105].

### 4.5. Novel and Experimental Therapies

Ruthenium complexes represent a new generation of metal anticancer drugs which have gained a lot of interest by the scientific community [106]. A series of novel lawsone-containing ruthenium (II) complexes have been recently synthetized and their antitumor effects have been investigated in DU145 spheroids. Among the tested agents, complex (4) exhibited remarkable anti-PCa activity, not only by impairing tumor cell growth but also by suppressing cancer cell adhesion and migration [107]. Overall, these data provide an example of how 3D PCa cell cultures can be used to elucidate the effects of new chemotherapeutic candidates on multiple tumor-related processes at once.

Proteasome inhibitors are a new group of potential antitumor drugs currently under investigation in early-phase clinical trials [108]. PS-341, a dipeptide boronic acid analogue, showed equal or greater proapoptotic activity in PCa tumoroids than in the respective monolayer cell cultures, even in the more slowly growing 3D models [109]. Therefore, these preliminary observations point out that this compound can circumvent the limited drug penetration observed with other common cytotoxic agents, supporting its encouraging effects against a wide spectrum of solid tumors.

The anticancer activity of pioglitazone, a common anti-diabetic agent, is still a matter of debate [110]. A preliminary study by Gottfried et al. revealed that it can reduce oxygen consumption and increase lactate secretion in 3D PC3 and LNCaP cell cultures, and that the combination with 2-deoxyglucose, a potent inhibitor of glycolysis, has an additive effect on cell proliferation inhibition and spheroid disintegration [111]. Globally, by using a tissue-like 3D setting, the authors propose a new anti-PCa mode of action for this drug via modulation of tumor cell metabolism.

The choice of a suitable delivery vector is crucial for the development of effective gene or RNA interference (RNAi) therapies [112,113]. In preliminary functional assays involving 3D PCa models, both cationic and cyclodextrin particles have successfully emerged as suitable carriers for plasmid DNA and siRNAs [114,115,116].

Antiangiogenic therapy is currently in use for treatment of different malignancies. The first anti-VEGF antibody described was the mouse monoclonal antibody A4.6.1, originally identified by its ability to block endothelial cell proliferation stimulated by recombinant human VEGF-A [117]. Interestingly, among the several experiments performed across the last two decades, a pilot study conducted by Borgström et al. showed that A4.6.1 treatment of DU145 spheroids implanted into dorsal skinfold chambers in nude mice was followed by complete inhibition of neovascularization of the microtumors as well as by suppression of tumor proliferation after the initial prevascular growth phase, thereby substantially contributing to current knowledge of antiangiogenic therapy benefits [118].

In recent years, many natural compounds have shown promise as anti-PCa agents [119,120,121,122,123]. Among them, an Arum Palaestinum-derived fraction enriched with isovanillin, linolenic acid, and β-sitosterol has been found to induce cell death in 3D PCa cell cultures [124]. Notably, these data are in agreement with those obtained in xenografted prostate tumors, underlying the strict correlation between 3D and in vivo models in terms of result reproducibility.

Collectively, several 3D cell culture assays have been employed for drug screening in PCa. The retention of the in vivo tumor characteristics as well as the addition of distinct tumor microenvironment components to PCa cells grown in 3D conditions have contributed to successfully identify differences in the anticancer activity of numerous agents. However, there is still an urgent need for the development of 3D models that not only recapitulate the essential elements of PCa but are also automated and analyzed in a high-throughput way. In this respect, it should be emphasized that novel microwell-based 3D culture platforms have been recently established to study PCa drug response [125,126,127,128].

## 5. Conclusions

To conclude, a deeper understanding of the complex mechanisms influencing PCa development and progression is essential to move closer towards tumor eradication in patients. Research into new 3D models that more closely reflect the tumor microenvironment in systems that allow drug screening practices is underway, with impressive progress published in the last decade. In particular, advances in biomaterials and micro-fabrication have enabled the rapid development of easy and inexpensive 3D PCa cell culture techniques. In addition, 3D co-culture systems with PCa cells and stromal, endothelial, or immune cells have been recently obtained, more effectively mimicking real cancer niches and thus helping clarify the interactions between the tumor and its microenvironment. In this context, 3D PCa cell cultures have shown great promise not only for disease simulation but also for drug discovery and tumor-targeted therapy testing. To this end, automated quantification of PCa spheroids has been proposed in order to achieve high throughput drug screening programs. In parallel, PDEs are under investigation as promising tools for preclinical drug development, paving the way towards personalized medicine for PCa. In the future, we expect 3D PCa models to be extensively used to validate preclinical outcomes, supporting animal experimentation and possibly facilitating its replacement.

## Figures and Tables

**Table 1:** Methods of 3D prostate cancer (PCa) cell culture.

Method	Advantages	Concerns	Ref.
Suspension cell cultures	Simple, low-cost, consistent yield, suitable for multicellular spheroids	Difficult control of spheroid size, lack of extracellular matrix surrogates, not suitable for migration/invasion or cell viability assays	[12,13,14,15,16,17,18,19]
Hanging drop	Low-cost, uniform spheroids	Labor-intensive, difficult medium exchange, lack of extracellular matrix surrogates, not suitable for migration/invasion or cell viability assays	[21]
Microfluidic devices	Uniform spheroids, control of spheroid size, fast spheroid formation, constant perfusion, uniform distribution of oxygen and nutrients	Specialized equipment and expertise, expensive, labor-intensive	[23]
Gel-embedding	Extracellular matrix-mimics, suitable for migration/invasion assays, wide variety of polymers	Undefined composition of natural gels, structural modification over time, labor-intensive	[24,25,26,27,28,29]
Scaffolds	High tissue mimics, wide variety of materials with wide variety of properties	Expensive, labor-intensive, possible variability between scaffolds	[30,31,32]
Patient-derived explants	High tissue mimics, direct assessment of patients’ therapeutic responses	Reliance on fresh tissue, specialized equipment and expertise, labor-intensive	[33,34,35,36,37,38]

**Table 2:** 3D PCa cell cultures in drug discovery and screening.

Treatment	Model	Outcomes	Ref.
Radiotherapy	PC3 cell spheroids (hanging drop)	Surface-Enhanced Raman Spectroscopy (SERS) can be used to assess treatment response	[89]
	LNCaP-LN3 cell spheroids (suspension)	Liposomes can deliver radiation to the central region of micrometastatic tumors	[90]
	LNCaP, PC3, and CWR22Rv1 cell spheroids (suspension)	C75 and 17-N-allylamino-17-demethoxy geldanamycin (17AAG) can sensitize cancer cells to ionizing radiation	[91,92]
Hormone therapy	LNCaP cell/fibroblast co-culture (hanging drop)	Fibroblasts can confer bicalutamide and enzalutamide resistance to cancer cells	[95]
Chemotherapy	LNCaP and PC3 cell spheroids (suspension)	Folic acid-coated polymersomes and micelles containing taxanes can increase drug cytotoxicity by reaching the core of the tumor	[97,98]
	LNCaP-LN3 cell spheroids (suspension)	Doxorubicin can suppress tumor growth	[100]
	DU145 cell spheroids (suspension)	3D spatial arrangement confers doxorubicin resistance to cancer cells via overexpression of P-glycoprotein	[101]
	DU145 cell spheroids (suspension)	Polymer-doxorubicin conjugates can enhance drug penetration in tumor tissues	[102]
Targeted therapies	PC3 cell spheroids (suspension)	PTEN/AKT/mTOR inhibitors can suppress tumor growth	[104,105]
	LNCaP cell spheroids (Matrigel)	Hyperpolarized magnetic resonance spectroscopy (HP-MRS) can be used to assess treatment response	[105]
Novel and experimental therapies	LNCaP, DU145 and PC3 cell spheroids (suspension); 22Rv1 cell spheroids (microfluidic device) PC3 cell spheroids (Matrigel); LNCaP, PC3, and TRAMP-C2 cell spheroids (scaffold)	Ruthenium complexes, proteasome inhibitors, pioglitazone, anti-VEGF antibodies, and nutraceuticals can suppress tumor growth Cationic and cyclodextrin particles are suitable carriers for gene or RNA interference (RNAi) therapies	[107,109,111,118,124] [114,115,116]
